# “IT SHOULDN’T BE LIKE THIS”: FAMILY CAREGIVERS NAVIGATING INSURANCE FOR FAMILY MEMBERS WITH DEMENTIA

**DOI:** 10.1093/geroni/igad104.1200

**Published:** 2023-12-21

**Authors:** Mikayla Gordon-Wexler, Deborah Watman, Jennifer Reckrey

**Affiliations:** Icahn School of Medicine at Mount Sinai, New York City, New York, United States; Icahn School of Medicine at Mount Sinai, New York City, New York, United States; Icahn School of Medicine at Mount Sinai, New York City, New York, United States

## Abstract

Family caregivers of individuals with dementia who are dually eligible for Medicaid and Medicare must navigate the health insurance landscape to meet the complex medical and home care needs of their family members. The goal of this study is to explore how family caregivers make decisions around insurance and how these choices affect care. Semi-structured interviews were conducted from June-January 2022 with 15 family caregivers of dually eligible individuals with dementia enrolled in home-based primary care in New York City. Interviews were analyzed using both deductive and inductive thematic coding. Participants described how social workers and other professionals helped navigate insurance choices, but no centralized system for obtaining information existed. Once insurance plans were set-up, participants described advocating on behalf of their family member within the constraints of current insurance plans rather than considering alternatives due to high perceived burden of changing plans. Obtaining adequate home care hours (which frequently required multiple appeals) was a particular challenge. Caregivers did not see integrated Medicare-Medicaid plans as providing any inherent benefit to navigating insurance. This study highlights the barriers facing family caregivers as they navigate insurance choices and the limited support available. These challenges may contribute to resistance to changing plans even when gaps in needed coverage exist, especially gaps related to inadequate home care. Robust professional support for family members both immediately after loved one’s dementia diagnosis and as the disease progresses could alleviate burden on caregivers and increase their capacity to get their family members needed medical and home care.

